# Association between magnesium depletion score and the prevalence of kidney stones in the low primary income ratio: a cross-sectional study of NHANES 2007–2018

**DOI:** 10.1097/JS9.0000000000001822

**Published:** 2024-06-14

**Authors:** Jiahao Wang, Yunfei Xiao, Yaqing Yang, Shan Yin, Jianwei Cui, Ke Huang, Jia Wang, Yunjin Bai

**Affiliations:** a Department of Urology, Institute of Urology, West China Hospital, Sichuan University; b Department of Respiratory and Critical Care Medicine, West China Hospital, Sichuan University, Chengdu; c Department of Urology, Affiliated Hospital of North Sichuan Medical College, Nanchong, People’s Republic of China

**Keywords:** association, cross-section, kidney stones, low primary ratio, magnesium depletion score

## Abstract

**Introduction:**

To explore the association between magnesium depletion score (MgDS) and the prevalence of kidney stones in the low primary income ratio (PIR).

**Method:**

A cross-sectional study was conducted using data from the National Health and Nutrition Examination Survey 2007–2018. Within the low PIR, people aged ≥20 years with complete information on MgDS and kidney stones questionnaires were enrolled. Multivariable logistic regression and stratified logistic regression analyses were performed to examine the association between MgDS and the prevalence of kidney stones and the recurrence of kidney stones by confounding factors adjusted. Stratified and interaction analysis was conducted to find whether some factors modified the association. In addition, sensitive analyses were also conducted to observe the stability. The work has been reported in line with the strengthening the reporting of cohort, cross-sectional, and case–control studies in surgery (STROCSS) criteria (Supplemental Digital Content 1, http://links.lww.com/JS9/C781).

**Result:**

A total of 7600 adults were involved in the study, and the individuals were classified into four groups: 0 points for MgDS (*n*=3814), 1 point for MgDS (*n*=2229), 2 points for MgDS (*n*=1020), and ≥3 points for MgDS (*n*=537). The multivariable logistic regression suggested that a positive association between MgDS and the prevalence of kidney stones (OR=1.123, 95% CI: 1.019–1.238) in the fully adjusted model. Compared with the lowest group, people with ≥3 points of MgDS had a significant relationship with kidney stones (OR=1.417, 95% CI: 1.013–1.983). No significant association was observed between the recurrence of kidney stones and MgDS. The result of the sensitive analysis showed the robustness of the main analysis.

**Conclusion:**

The prevalence of kidney stones is positively correlated with MgDS, which suggests that maintaining a higher MgDS is accompanied by higher prevalence rates of kidney stones in the low PIR.

## Introduction

Highlights

- Adequate magnesium intake is associated with a decreased risk of stone formation by inhibiting crystal aggregation and reducing the supersaturation of stone-forming compounds in the urine.
- Magnesium depletion score (MgDS) outperformed assessments based on serum and urinary magnesium concentrations in diagnosing magnesium deficiency.
- There is a positive association between MgDS and the prevalence of kidney stones in the low primary income ratio.
- No significant correlation was observed between the recurrence of kidney stones and MgDS within a low-income demographic.
- Tailored dietary interventions and social support initiatives be implemented for low-income populations to enhance magnesium levels and mitigate the risk of kidney stone formation.

Kidney stones have become increasingly prevalent in the field of urology, posing a significant concern due to rising medical expenses and societal impact. A nationwide study indicated that the occurrence of kidney stones varies from 1.7 to 14.8%, with rates consistently increasing annually worldwide^1^. Notably, the high recurrence rate, with ~30% of patients experiencing a relapse within 5 years of the initial episode, places a considerable strain on healthcare systems^2^. In the United States, the annual cost of kidney stone treatment surpasses $5 billion, with removal expenses outweighing preventive measures^3^. Consequently, while treatment methods for kidney stones have advanced, prioritizing prevention remains paramount for cost-effectiveness. Despite the identification of various chronic conditions as risk factors for kidney stones, the precise underlying mechanism remains incompletely understood. Therefore, there is an urgent need for a comprehensive exploration of preventive strategies to address the issue of kidney stones.

Recent studies show that some dietary trace metals act as protect roles for kidney stones, such as copper, phosphorus, and magnesium^4^. Magnesium is the second most abundant intracellularly, following potassium, acting as a natural antagonist to calcium oxalate and phosphate crystal formation, the most common constituents of kidney stones^5^. Adequate magnesium intake is associated with a decreased risk of stone formation by inhibiting crystal aggregation and reducing the supersaturation of stone-forming compounds in the urine^6^. Despite its significance, over half of adults in America fail to consume sufficient magnesium^7^. Economic constraints in certain communities, such as a low primary income ratio (PIR), can greatly restrict the ability to obtain magnesium-rich foods. This, combined with dietary deficiencies or genetic influences, can raise the likelihood of developing magnesium deficiency and, as a result, kidney stone formation^8^. A prolonged lack of magnesium intake could result in chronic or hidden magnesium deficiency^9^. Nevertheless, the absence of noticeable clinical symptoms or signs often accompanies magnesium deficiency, and there is a lack of standardized tests to precisely evaluate magnesium levels. While the magnesium tolerance test is widely considered the most reliable approach for assessing magnesium status, its clinical utility is restricted due to the necessity of two distinct 24 h urine collections before and after a 4 h intravenous magnesium infusion^10^. As a result, the magnesium depletion score (MgDS) offers a more precise and dependable alternative, taking into account four key factors: present intake of diuretics, current usage of proton pump inhibitors (PPIs), deterioration in kidney function, and level of alcohol consumption^11–13^. Furthermore, MgDS outperformed assessments based on serum and urinary magnesium concentrations in diagnosing magnesium deficiency^12^.

To our knowledge, there is no study focused on the association between MgDS and the prevalence of kidney stones. Given the socioeconomic disparities in health outcomes, understanding the link between magnesium depletion and kidney stones in low-income populations can inform targeted prevention and intervention strategies. Thus, this study aims to elucidate the association between MgDS and the prevalence of kidney stones among individuals with a low PIR, utilizing data from NHANES 2007–2018. Our conjecture suggests a correlation between elevated MgDS levels and a heightened incidence of kidney stones within this demographic, underscoring the significance of magnesium in dietary recommendations, and public health strategies aimed at mitigating kidney stone susceptibility.

## Method

### Study design and population

**Figure 1:**
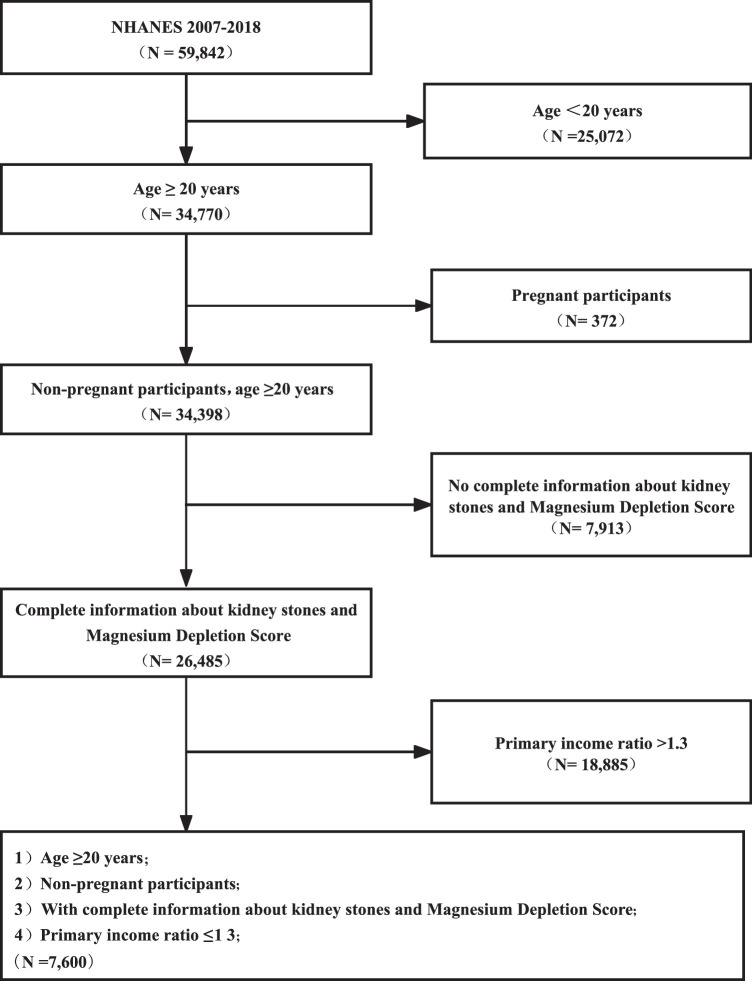
Flow diagram of obtaining the final inclusion in the population. Data available in a publicly accessible repository that does not issue DOIs. Publicly available datasets were analyzed in this study. These data can be found here: https://www.cdc.gov/nchs/nhanes/index.htm.

The NHANES provides prevalence estimates for an array of common diseases by performing a complex, multistage, probability sampling design. The data set in the NHANES database is typically updated periodically, with new waves of data released approximately every 2 years. The information utilized in this study was obtained from the NHANES database spanning from 2007 to 2018 (for the present analysis, six survey cycles (i.e. 2007–2008, 2009–2010, 2011–2012, 2013–2014, 2015–2016, and 2017–2018) were combined to produce estimates with greater precision and smaller sampling error), a project established by the National Center for Health Statistics (NCHS) that undergoes biennial updates starting from 1999. NHANES serves as a comprehensive and nationally representative survey focusing on the health and dietary habits of individuals within the United States. Through a multistage probability sampling approach, data is gathered via structured interviews, physical check-ups, and laboratory evaluations. With the primary aim of reflecting the demographics of the US population, stringent measures are in place during data collection to safeguard the privacy and confidentiality of participants. The initial sample collected from six continuous cycles totaled 59 842 individuals. Firstly, people younger than 20 years were not considered (*n*=34 770). Secondly, pregnancy was excluded (*n*=372). In addition, participants without complete information about MgDS and kidney stones were also removed (n=7913). The individuals with low PIR (PIR <1.3) were also not enrolled. Ultimately, there are 7600 adults admitted and the exclusion criteria are described in Figure 1.

In assessing our sample size adequacy, we followed the European Association of Urology (EAU) guidelines, which indicate a 10% prevalence rate of kidney stones in the United States. With a significance level (*α*) set at 0.01, we applied the formula $:n=\frac{Z_{1-a/2}^{2}*\mathrm{pq}}{d^{2}}\;$ to calculate the necessary sample size, where *Z* represents the *Z*-score for the chosen confidence level, *p* is the prevalence rate (q=1−p), and *d* is the margin of error. Based on our computations, we determined that a sample size of 5991 participants would be adequate. However, our final sample size consisted of 7600 individuals, surpassing the calculated requirement and ensuring the reliability and robustness of our findings.

All NHANES study protocols were approved by the Ethics Review Committee of the NCHS, and consent was obtained from all participants. This cross-sectional study was performed in line with the STROCSS 2021 and STROCSS 2021 Guidelines^14^.

### Outcomes and exposure variable

MgDS, a clinical composite index devised to assess magnesium deficiency within the body, is calculated by summing points from the four risk factors^12^. 1 diuretics use: ‘yes’ scores one point, ‘no’ scores zero points; 2 PPIs use: ‘yes’ scores one point, ‘no’ scores zero points; 3 kidney function: estimated glomerular filtration rate (eGFR) ≥90 scores zero point. eGFR <90 and ≥60 ml/min/1.73 m^2^ scores one point. eGFR <60 ml/min/1.73 m^2^ scores two points^15^; 4 heavy alcohol consumption, defined as more than two drinks per day for men and more than one drink per day for women, scores one point. All other drinking habits (never, ever, mild, and moderate) score zero points^16^. Each aspect plays a role in determining the complete score, showcasing the seriousness of magnesium insufficiency. Owing to the small sample of MgDS^3–5^, MgDS classifies people into four groups: 0, 1, 2, and ≥3, respectively. The primary outcome of the study was the prevalence of kidney stones. Data from the questionnaire can be extracted to calculate the prevalence of kidney stones. In the questionnaire, participants were categorized as having kidney stones if they responded affirmatively to the question, ‘Have you ever had kidney stones?’ Conversely, participants who replied negatively were assumed to be free of kidney stones.

### Other covariates

Continuous variables were age, BMI, fiber intake, fat intake, magnesium intake, calcium intake, energy intake, healthy eating index-2015 (HEI-2015), sedentary intake, and water intake. Categorical variables included sex (female and male), race (Mexican American, non-Hispanic white, non-Hispanic black, other Hispanic, other races), education (less than 9th grade, 9–11th grade, high school graduate, some college, college graduate or above), marital status (married/living with a partner, divorced/separated/widowed, never married), alcohol (never, former, mild, moderate, and heavy), smoking (never, former, and now), diabetes (no, borderline, and yes), stroke, cardiovascular disease (CVD), hypertension (HBP), moderate activity, and vigorous activity (all no/yes). In addition, sedentary time and calcium intake were divided into two groups based on the cut point of 150 mins/day and 300 mg/day. Adequate water required ≥2000 ml/day for females and 2500 ml/day for males.

### Statistical analysis

The study provided baseline characteristics in the form of means±SD for continuous variables and proportions for categorical variables. Correlations between continuous variables were determined using linear regression, and comparisons of categorical variables were adjusted using chi-square analysis.

Multivariate logistic regression analyses were employed to evaluate the potential association between MgDS and the prevalence of kidney stones with three logistic regression models conducted. In addition, another three logistic regression models were utilized to investigate the relationship between MgDS and recurrent kidney stones (people who had developed kidney stones more than once). In the nonadjusted model, no factor was adjusted. The minimally adjusted model was adjusted for age and race. The fully adjusted model was further adjusted for age, sex, race, education, marital status, BMI, alcohol, smoking, stroke, CVD, HBP, diabetes, moderate activity, vigorous activity, magnesium intake, calcium intake, fat intake, fiber intake sedentary time, water intake, energy intake, and HEI-2015. In order to identify whether some factors modify the association, the stratified and interaction analyses were made. The dietary information was gathered through 24 h recall interviews for the study. Water intake, considered to be a significant factor in kidney stone development, was specifically obtained via telephone 3–10 days after the initial interview, and sensitive analyses were conducted with adjustments made for water intake once again.

All the above statistical analyses were completed using R 4.2.2 (http://www.R-project.org, the R Foundation) and EmpowerStats (http://www.empowerstats.com, X&Y Solutions, Inc.). Statistical significance was set at *P*<0.05.

## Result

### Baseline characteristics of study participants

There were 7600 people enrolled in the study. The individuals were classified into four groups: 0 points for MgDS (*n*=3814), 1 point for MgDS (*n*=2229), 2 points for MgDS (*n*=1020), and ≥3 points for MgDS (*n*=537). The baseline characteristics showed significant differences among the four groups (Table 1). People with ≥3 MgDS were more likely to be female (62.76%) and older (67.42±11.00 years), have higher BMI (32.55±8.12 kg/m^2^), and have longer sedentary time (394.83±207.55 mins/day). In addition, the highest proportion of kidney stones (16.57%), low-educated (below high school graduate) (71.71%), stroke (18.25%), CVD (43.58%), HBP (91.25%), diabetes (52.14%) were indicated in this group. Conversely, the lowest magnesium intake (237.68±97.61 mg/day) and calcium intake (777.29±439.30 mg/day) and energy intake (1684.92±778.84 kcal), and the lowest proportion of moderate activity (18.99%) and vigorous activity (2.79%) and never married (9.12%). In addition, compared to the other three groups, people with 0 points MgDS prefer to never smoke (55.61%) and never alcohol (19.34%) and have the highest fiber intake (16.32±9.73 mg/day) and fat intake (76.08±40.74 mg/day) and lowest HEI-2015 (47.57±13.51). Compared to nonkidney stones people, participants with kidney stones were more likely to have higher MgDS and lower magnesium intake (Supplementary Table S1, Supplemental Digital Content 2, http://links.lww.com/JS9/C782).

**Table 1:** Characteristics of participants with low PIR by magnesium depletion score: NHANES 2007–2018.^a^

		MgDS				
Variables	All (*n*=7600)	0 (*n*=3814)	1 (*n*=2229)	2 (*n*=1020)	≥3 (*n*=537)	*P*
MgDS (mean±SD)	0.79±0.97	0.00±0.00	1.00±0.00	2.00±0.00	3.20±0.42	<0.001
Kidney stones (%)						<0.001
No	90.45	92.58	90.13	86.86	83.43	
Yes	9.55	7.42	9.87	13.14	16.57	
Age (years, mean±SD)	47.84±17.87	38.85±14.22	51.49±16.70	63.16±13.50	67.42±11.00	<0.001
20–34 (%)	28.87	45.65	18.75	3.33	0.19	
35–49 (%)	25.18	30.49	25.89	13.14	7.45	
50–64 (%)	24.49	18.56	29.97	32.25	29.05	
≥65 (%)	21.46	5.30	25.39	51.27	63.31	
PIR (mean±SD)	0.79±0.35	0.76±0.35	0.81±0.35	0.84±0.32	0.89±0.30	<0.001
BMI (kg/m^2^, mean±SD)	29.87±7.63	29.37±7.50	29.62±7.44	30.92±7.91	32.55±8.12	<0.001
<25 (%)	26.94	29.83	27.26	22.33	13.35	
≥25 and <30 (%)	31.11	30.78	32.88	29.07	29.98	
≥30 (%)	41.94	39.40	39.86	48.59	56.67	
Sex (%)						<0.001
Female	55.32	57.03	50.56	55.39	62.76	
Male	44.68	42.97	49.44	44.61	37.24	
Race (%)						<0.001
Mexican American	19.92	25.33	15.79	13.43	10.99	
Other Hispanic	37.11	30.39	42.04	46.08	47.30	
Non-Hispanic white	23.00	21.97	22.88	24.41	28.12	
Non-Hispanic black	12.03	13.50	11.04	10.10	9.31	
Other races	7.95	8.81	8.25	5.98	4.28	
Education (%)						<0.001
Less than 9th grade	16.63	15.32	15.94	20.59	21.27	
9–11th grade	22.28	22.59	21.60	22.16	23.13	
High school graduate	26.58	26.65	27.30	24.90	26.31	
Some college	26.35	26.63	26.36	25.98	25.00	
College graduate or above	8.16	8.81	8.80	6.37	4.29	
Marital (%)						<0.001
Married/Living with partner	47.01	51.07	45.94	40.00	35.94	
Divorced/Separated/Widowed	29.86	19.14	33.92	47.84	54.93	
Never married	23.13	29.79	20.14	12.16	9.12	
Alcohol (%)						<0.001
Never	17.74	19.34	14.44	19.08	17.72	
Former	20.87	16.85	20.21	27.87	38.29	
Yes	61.39	63.81	65.35	53.05	43.99	
Smoke (%)						<0.001
Never	48.43	55.61	41.34	41.18	40.60	
Former	21.39	14.61	23.34	32.16	40.97	
Yes	30.18	29.77	35.32	26.67	18.44	
Stroke (%)						<0.001
No	94.58	97.66	94.88	89.16	81.75	
Yes	5.42	2.34	5.12	10.84	18.25	
HBP (%)						<0.001
No	55.32	73.20	50.83	22.75	8.75	
Yes	44.68	26.80	49.17	77.25	91.25	
CVD (%)						<0.001
No	86.33	94.57	86.68	70.46	56.42	
Yes	13.67	5.43	13.32	29.54	43.58	
Diabetes (%)						<0.001
No	70.16	78.92	70.17	54.12	38.36	
Borderline	8.46	7.97	8.93	8.73	9.50	
Yes	21.38	13.11	20.91	37.16	52.14	
Water intake (%)	1019.50±1215.76	1075.54±1252.84	1005.88±1238.28	885.82±1089.59	932.00±1045.77	<0.001
Inadequate	85.88	84.32	86.09	89.41	89.39	
Adequate	14.12	15.68	13.91	10.59	10.61	
Moderate activity						<0.001
No	68.93	66.19	68.13	74.51	81.01	
Yes	31.07	33.81	31.87	25.49	18.99	
Vigorous activity						<0.001
No	85.18	79.94	87.03	94.41	97.21	
Yes	14.82	20.06	12.97	5.59	2.79	
Sedentary time (%)	323.69±199.22	302.42±195.13	329.66±197.65	352.91±200.34	394.83±207.55	<0.001
<150 mins/day	20.50	23.76	19.14	15.88	11.65	
≥150 mins/day	79.50	76.24	80.86	84.12	88.35	
Calcium intake (%)	885.31±517.24	922.41±534.18	889.82±530.82	793.57±435.87	777.29±439.30	<0.001
<300 mg/day	5.84	5.11	5.74	7.55	8.19	
≥300 mg/day	94.16	94.89	94.26	92.45	91.81	
Fiber intake (mg/day, mean±SD)	15.51±9.18	16.32±9.73	15.20±9.05	14.20±7.79	13.54±7.27	<0.001
Fat intake (mg/day, mean±SD)	73.54±39.84	76.08±40.74	74.50±41.16	67.82±36.13	62.43±30.61	<0.001
Magnesium intake (mg/day, mean±SD)	269.86±128.14	273.08±130.72	280.10±133.70	252.40±115.31	237.68±97.61	<0.001
Energy intake (kcal, mean±SD)	2063.28±1080.28	2122.95±1082.82	2138.99±1160.68	1873.92±955.80	1684.92±778.84	<0.001
HEI-2015 (mean ±SD)	48.69±13.44	47.57±13.51	49.93±13.23	49.56±13.52	49.90±12.92	<0.001

^a^ Mean+SD for continuous variables, and *P*-value was calculated by weighted *t*-test. % for categorical variables, and *P*-value was calculated by weighted χ^2^ test.

CVD, cardiovascular disease; HBP, hypertension; HEI-2015 Index, healthy eating index-2015; MgDS, magnesium depletion score; PIR, poverty income ratio.

### Multivariate regression analysis

The multivariable regression analyses showed that a positive association between MgDS and the prevalence of kidney stones in the nonadjusted model (OR=1.341, 95% CI: 1.249–1.439, *P*<0.001), minimally-adjusted model (OR=1.202, 95% CI: 1.102–1.311, *P*<0.001), fully adjusted model (OR=1.123, 95% CI: 1.019–1.238, *P*<0.001) (Table 2). Subgroup analyses indicated that 3 points of MgDS had a significant relationship with kidney stones in the fully adjusted model (OR=1.417, 95% CI: 1.013–1.983, *P*=0.042). Moreover, no significant association was found between the recurrence of kidney stones and MgDS in three models (Table 3).

**Table 2:** Association between MgDS and the prevalence of kidney stones for the low PIR.

	Nonadjusted model^a^		Minimally-adjusted model^b^		Fully adjusted model^c^	
Variables (%)	OR (95% CI)	*P*	OR (95% CI)	*P*	OR (95% CI)	*P*
MgDS	1.341 (1.249–1.439)	<0.001	1.202 (1.102–1.311)	<0.001	1.123 (1.019–1.238)	0.020
Categories of MgDS						
0	Ref		Ref		Ref	
1	1.366 (1.136–1.643)	<0.001	1.092 (0.895–1.332)	0.385	1.150 (0.928–1.425)	0.201
2	1.887 (1.517–2.347)	<0.001	1.374 (1.069–1.767)	0.013	1.242 (0.942–1.637)	0.124
≥3	2.479 (1.916–3.207)	<0.001	1.809 (1.344–2.433)	<0.001	1.417 (1.013–1.983)	0.042

OR, odds ratio.

^a^ Nonadjusted model adjusts for none.

^b^ Minimally adjusted model adjusts for age, race.

^c^ Fully adjusted model adjusts for age, sex, BMI, race, education, marital, alcohol, smoke, diabetes, hypertension, cardiovascular disease, stroke, energy intake, healthy eating index-2015, sedentary time, vigorous activity, moderate activity, water intake, calcium intake, magnesium intake, fiber intake, fat intake.

**Table 3:** Association between MgDS and the recurrence of kidney stones for the low PIR.

	Nonadjusted model^a^		Minimally-adjusted model^b^		Fully adjusted model^c^	
Variables (%)	OR (95% CI)	*P*	OR (95% CI)	*P*	OR (95% CI)	*P*
MgDS	0.947 (0.773–1.161)	0.602	1.017 (0.787–1.314)	0.899	1.098 (0.811–1.485)	0.546
Categories of MgDS						
0	Ref		Ref		Ref	
1	1.350 (0.754–2.418)	0.313	1.549 (0.813–2.949)	0.183	1.706 (0.813–3.581)	0.158
2	1.165 (0.616–2.202)	0.639	1.572 (0.729–3.387)	0.249	2.195 (0.924–5.214)	0.075
≥3	0.850 (0.416–1.737)	0.656	1.160 (0.486–2.766)	0.739	1.345 (0.472–3.832)	0.580

OR, odds ratio.

^a^ Nonadjusted model adjusts for none.

^b^ Minimally adjusted model adjusts for age, race.

^c^ Fully adjusted model adjusts for age, sex, BMI, race, education, marital, alcohol, smoke, diabetes, hypertension, cardiovascular disease, stroke, energy intake, healthy eating index-2015, sedentary time, vigorous activity, moderate activity, water intake, calcium intake, magnesium intake, fiber intake, fat intake.

### Stratified and interaction analysis

To identify whether some factors modify the association between MgDS and the prevalence of kidney stones, stratified and interaction analyses were conducted. Table 4 showed that BMI and calcium intake had some significant effects on the association. The three groups of BMI stratified by 25 and 30 kg/m^2^ cut points contributed the association more obvious (BMI <25 kg/m^2^ OR=1.378, BMI ≥25 and <30 kg/m^2^ OR=1.018, BMI ≥30 kg/m^2^ OR=1.119). Moreover, both of the stratified calcium intake showed the same trend (<300 mg/day OR=1.604, ≥300 mg/day OR=1.097).

**Table 4:** Logistic regression analysis to identify variables that modify the correlation between MgDS and the prevalence of kidney stones for the low PIR.

	Fully adjusted model^a^		
	OR (95% CI)		
Variables (%)	Food security	Food insecurity	*P* for interaction
Age (years)			0.923
20–34	Ref	1.209 (0.791–1.846)	
35–49	Ref	1.109 (0.910–1.351)	
50–64	Ref	1.085 (0.928–1.269)	
≥65	Ref	1.158 (0.992–1.352)	
BMI (kg/m^2^)			0.042
<25	Ref	1.378 (1.129–1.681)	
≥25 and <30	Ref	1.018 (0.870–1.191)	
≥30	Ref	1.119 (0.992–1.262)	
Sex			0.312
Female	Ref	1.083 (0.960–1.222)	
Male	Ref	1.175 (1.031–1.339)	
Race			0.523
American	Ref	1.144 (0.922–1.421)	
Non-Hispanic white	Ref	1.069 (0.945–1.210)	
Non-Hispanic black	Ref	1.311 (1.072–1.603)	
Hispanic	Ref	1.111 (0.874–1.412)	
Other races	Ref	1.100 (0.782–1.549)	
Education			0.078
Less than 9th grade	Ref	1.325 (1.108–1.584)	
9–11th grade	Ref	1.203 (1.011–1.432)	
High school graduate	Ref	0.997 (0.837–1.187)	
Some college	Ref	1.013 (0.861–1.192)	
College graduate or above	Ref	1.245 (0.879–1.763)	
Marital			0.603
Married/Living with partner	Ref	1.099 (0.963–1.254)	
Divorced/Separated/Widowed	Ref	1.119 (0.979–1.277)	
Never married	Ref	1.268 (0.979–1.641)	
Alcohol			0.103
Never	Ref	1.299 (1.071–1.575)	
Former	Ref	1.178 (1.009–1.376)	
Now	Ref	1.043 (0.921–1.182)	
Smoke			0.992
Never	Ref	1.130 (0.985–1.295)	
Former	Ref	1.117 (0.958–1.302)	
Now	Ref	1.121 (0.951–1.321)	
Diabetes			0.661
No	Ref	1.166 (1.027–1.323)	
Borderline	Ref	1.070 (0.805–1.423)	
Yes	Ref	1.083 (0.940–1.246)	
HBP			0.271
No	Ref	1.221 (1.024–1.456)	
Yes	Ref	1.094 (0.982–1.219)	
CVD			0.688
No	Ref	1.110 (0.990–1.244)	
Yes	Ref	1.154 (0.980–1.358)	
Stroke			0.846
No	Ref	1.120 (1.011–1.240)	
Yes	Ref	1.153 (0.866–1.536)	
Moderate activity			0.842
No	Ref	1.118 (1.005–1.244)	
Yes	Ref	1.139 (0.960–1.352)	
Vigorous activity			0.388
No	Ref	1.114 (1.008–1.230)	
Yes	Ref	1.295 (0.928–1.807)	
Sedentary time (%)			0.721
<150 mins/day	Ref	1.163 (0.939–1.440)	
≥150 mins/day	Ref	1.116 (1.007–1.237)	
Water intake (%)			0.425
Inadequate	Ref	1.107 (0.998–1.228)	
Adequate	Ref	1.210 (0.985–1.486)	
Calcium intake (%)			0.020
<300 mg/day	Ref	1.604 (1.170–2.200)	
≥300 mg/day	Ref	1.097 (0.992–1.212)	

OR, odds ratio.

^a^ Fully adjusted model adjusts for age, sex, BMI, race, education, marital, alcohol, smoke, diabetes, hypertension, cardiovascular disease, stroke, energy intake, healthy eating index-2015, sedentary time, vigorous activity, moderate activity, water intake, calcium intake, magnesium intake, fiber intake, fat intake.

### Sensitivity analysis

The multivariable logistic regressions were conducted with water intake based on day 2 dietary recall data adjusted. Supplementary Table S2 (Supplemental Digital Content 3, http://links.lww.com/JS9/C783) showed that MgDS was positively associated with the prevalence of kidney stones, and subgroup analysis supported that ≥3 points of MgDS are positively related to kidney stones. In addition, Supplementary Table S3 (Supplemental Digital Content 4, http://links.lww.com/JS9/C784) showed no significant association between the recurrence of kidney stones and MgDS, which was consistent with the previous.

## Discussion

This research investigation examined the relationship between MgDS and the prevalence of kidney stones through the analysis of NHANES data spanning from 2007 to 2018. The findings indicated a positive association between MgDS and the prevalence of kidney stones, particularly in the fully model following adjustments for multiple variables, with this association maintaining significance. This correlation was particularly pronounced in individuals with elevated MgDS (≥3 points). However, no significant correlation was observed between the prevalence of recurrent kidney stones and MgDS. Furthermore, our study revealed that both BMI and calcium intake exerted a noteworthy positive moderating influence on the association between MgDS and the prevalence of kidney stones. Sensitivity analysis further validated the robustness of these findings.

Magnesium is a crucial factor in the prevention of kidney stone formation, as supported by existing research. Various clinical and fundamental investigations have demonstrated that magnesium has the ability to create a soluble compound with oxalic acid, thereby diminishing the deposition of calcium oxalate through the inhibition of exogenous oxalic acid absorption in the intestines, resulting in decreased levels of urinary oxalate and urinary supersaturation^17–19^. Moreover, magnesium has been shown to inhibit the crystallization and growth of calcium oxalate by extending the induction period of calcium oxalate crystals administration of magnesium supplements has been shown to induce anti-inflammatory reactions and diminish the presence of proinflammatory indicators resulting from magnesium deficiency^20,21^. Consequently, a significant correlation exists between magnesium and crystals development^22^. Additionally, the consumption of magnesium salts can elevate urine pH, enhance the excretion of citric acid, and subsequently mitigate the development of kidney stones^23^. However, the acidification of urine has been found to expedite the loss of magnesium^24^. Research indicates that magnesium deficiency is linked to mitochondrial dysfunction, abnormal levels of oxidative stress markers (such as plasma superoxide anions and malondialdehyde, GSH, SOD, MDA), impaired DNA repair capacity, and heightened genomic instability, all of which are factors that elevate the likelihood of developing kidney stones. This suggests a potential mechanism for stone formation^25^. Research conducted on animals has shown that a magnesium-deficient diet leads to an imbalance in antioxidant and oxidant levels within the body, impaired redox ability, and heightened susceptibility to lipid peroxidation damage^26^. Magnesium deficiency exacerbates the sensitivity of vascular endothelial cells to oxidative stress induced by hydrogen peroxide, resulting in increased free radical-induced damage and cytotoxicity. These findings provide insight into the potential mechanism by which magnesium deficiency may contribute to an elevated risk of kidney stone formation^27,28^. On the contrary, there is magnesium depletion and heightened susceptibility to kidney stone formation.

Indirect evidence indicates that magnesium depletion may elevate the likelihood of multiple distinct risk factors associated with the development of kidney stones, consequently contributing to an increased prevalence of kidney stone formation. Moreover, magnesium deficiency is closely linked to various components of metabolic syndrome, including insulin resistance, HBP, and obesity, further heightening the risk of CVD, all of which collectively amplify the susceptibility to kidney stone formation^29–33^. Magnesium is integral to numerous biological processes implicated in the pathogenesis of various diseases, including insulin signaling, blood pressure regulation, and lipid metabolism^34–36^. Factors such as the presence of components of metabolic syndrome and the use of certain medications (e.g. diuretics, PPIs) can exacerbate magnesium loss and hinder its absorption, thereby heightening the risk of magnesium deficiency^37–39^. Thus, a negative feedback loop is present. Moreover, the precise mechanisms are not yet fully understood, but magnesium deficiency frequently coexists with heightened uric acid levels, thereby heightening the likelihood of uric acid kidney stone development^40,41^. So, the depletion of magnesium may indirectly facilitate the formation of kidney stones by impacting various metabolic pathways within the organism.

Magnesium deficiency is primarily influenced by dietary magnesium intake and excretion levels. Inadequate magnesium intake is prevalent among the general population, with research indicating that individuals of lower socioeconomic status are at a heightened risk of magnesium depletion. This susceptibility may be attributed to factors such as suboptimal dietary habits, limited health literacy, and restricted availability of nutritious food options^42,43^. Research has shown that individuals with lower income levels are more likely to have limited access to magnesium-rich foods, such as green leafy vegetables, nuts, and whole grains, which can contribute to an increased risk of magnesium deficiency^8,44^. Furthermore, financial strain and a lower socioeconomic status may also heighten the likelihood of engaging in unhealthy behaviors, such as consuming a high-fat diet, smoking, and leading a sedentary lifestyle, all of which are recognized risk factors for the development of kidney stones^45,46^. Hence, it is imperative to implement targeted interventions and policies aimed at improving the nutritional status of magnesium among low-income populations and addressing the specific needs of vulnerable groups in order to mitigate the risk of kidney stone formation. For example, the eligibility criteria for the federal and state joint Medicaid programs that provide health insurance to millions of low-income Americans should be appropriately relaxed. Actively advancing the Children’s Health Insurance Program (CHIP) and Affordable Care Act (ACA) marketplaces to offer low-cost health insurance to children in families with incomes too high to qualify for Medicaid but unable to afford private insurance, while low-income individuals may qualify for subsidies to help offset premiums and out-of-pocket costs. Establishing federally qualified health centers (FQHCs) and community health centers to provide primary healthcare services to underserved and low-income populations, including medical, dental, and behavioral health services. Operating free and low-cost clinics by nonprofit organizations or local governments to offer basic medical services at reduced or no cost to uninsured and low-income individuals. Encouraging many pharmaceutical companies to provide prescription assistance programs to help eligible low-income individuals with discounts or waivers of medication costs. Most importantly, vigorously promoting supplemental nutrition assistance programs (SNAP) and Women, Infants, and Children (WIC) programs to help low-income individuals and families access nutritious foods, which can have a positive impact on their overall health. These initiatives not only increase the prevention and treatment possibilities for urinary stones but also significantly improve the health-related quality of life for citizens.

This study demonstrates the significant impact of calcium intake and BMI on the association between magnesium levels and the formation of kidney stones. It is advised to maintain a balanced calcium intake as the primary dietary measure for preventing kidney stones. However, both excessive and inadequate calcium intake may elevate the risk of kidney stone formation, particularly when magnesium intake is insufficient^47–49^. The stratified analysis of calcium intake revealed a correlation between low calcium intake (<300 mg/day) and an elevated risk of kidney stones. This association may be attributed to the potential mechanism by which a low calcium diet leads to an increase in free oxalate ions in the intestine, facilitating their absorption and subsequently raising urinary oxalate excretion levels^50^. Furthermore, obesity, defined as a BMI of 30 kg/m^2^ or higher, is recognized as an autonomous risk factor for the development of kidney stones. Our research substantiates that the association between magnesium dietary supplement (MgDS) intake and kidney stone risk varies across different BMI categories. The elevated risk of kidney stones in individuals with obesity may be attributed to alterations in urine composition, such as decreased urine pH and heightened excretion of calcium and oxalate^51,52^. Therefore, the interaction between calcium intake and BMI may influence the relationship between magnesium and kidney stones through multiple mechanisms.

This article provides a significant contribution by examining the association between magnesium consumption scores and kidney stones in low-income individuals for the first time, utilizing a large multiethnic sample population from NHANES to enhance the reliability and generalizability of the research findings. However, limitations exist in the study design. As it is a cross-sectional study, preventing the establishment of a causal relationship between the variables. Secondly, the data collection of kidney stones is completed through interviews. Although it is conducted by trained professionals, recall bias is inevitable. Information on the composition and size of kidney stones is absent in the database, which limits our further research.

## Result

This study identified a notable correlation between magnesium intake levels and the occurrence of kidney stones within a low-income demographic. So, it is important to recommend that tailored dietary interventions and social support initiatives be implemented for low-income populations to enhance magnesium levels and mitigate the risk of kidney stone formation. In addition, the comprehensive and longitudinal investigations are supposed to delve deeper into the underlying mechanisms of this relationship.

## Ethical approval

This study was performed using public data from the National Center for Health Statistics (NCHS) program and the National Health and Nutrition Examination Survey (NHANES). The data have been de-identified and not merged or augmented in a way that has compromised the privacy of the participants. Therefore, the study requires no further approval and follows ethical guidelines. In addition, participant data were obtained from the publicly available NHANES, so no additional consent was obtained.

## Consent

This study was performed using public data from the National Center for Health Statistics (NCHS) program and the National Health and Nutrition Examination Survey (NHANES). The data have been de-identified and not merged or augmented in a way that has compromised the privacy of the participants. Therefore, the study requires no further approval and follows ethical guidelines. In addition, participant data were obtained from the publicly available NHANES, so no additional consent was obtained.

## Source of funding

This work was supported by the Key Research and Development Projects of Sichuan Science and Technology Department (grant numbers: 2022YFS0306).

## Author contribution

J.W. and J.H.W.: conception and design; J.W.: administrative support and supervision; J.H.W., Y.F.X., Y.Q.Y., S.Y., J.W.C., and K.H.: collection and assembly of data; Y.F.X., Y.Q.Y., and Y.J.B.: data analysis and interpretation; J.H.W. and Y.F.X.: manuscript writing; All authors contributed in final approval of the manuscript.

## Conflicts of interest disclosure

All authors certify that they have no affiliations with or involvement in any organization or entity with any financial interest or non-financial interest in the subject matter or materials discussed in this manuscript.

## Research registration unique identifying number (UIN)

1. Name of the registry: not applicable.
2. Unique identifying number or registration ID: not applicable.
3. Hyperlink to your specific registration (must be publicly accessible and will be checked): not applicable.

## Guarantor

Jia Wang. Department of Urology, Institute of Urology, West China Hospital, Sichuan University, No. 37, Guoxue Alley, Chengdu, Sichuan, People’s Republic of China. E-mail: wangjiawch@163.com (JW) Yunjin Bai. Department of Urology, Institute of Urology, West China Hospital, Sichuan University, No. 37, Guoxue Alley, Chengdu, Sichuan, People’s Republic of China. E-mail: baiyunjin@163.com (YJB).

## Data availability statement

Data available in a publicly accessible repository that does not issue DOIs. Publicly available datasets were analyzed in this study. These data can be found here: https://www.cdc.gov/nchs/nhanes/index.htm.

## Provenance and peer review

Not commissioned, externally peer-reviewed.

## Supplementary Material

SUPPLEMENTARY MATERIAL

## References

[1] Romero V Akpinar H Assimos DG. Kidney stones: a global picture of prevalence, incidence, and associated risk factors. Rev Urol 2010;12:e86–e96. 20811557 PMC2931286

[2] Kittanamongkolchai W Vaughan LE Enders FT. The changing incidence and presentation of urinary stones over 3 decades. Mayo Clin Proc 2018;93:291–299. 29452705 10.1016/j.mayocp.2017.11.018 PMC5849397

[3] Alelign T Petros B. Kidney stone disease: an update on current concepts. Adv Urol 2018;2018:3068365. 29515627 10.1155/2018/3068365 PMC5817324

[4] Zhu W Wang C Wu J. Dietary copper intake and the prevalence of kidney stones among adult in the United States: a propensity score matching study. Front Public Health 2022;10:973887. 36111192 10.3389/fpubh.2022.973887 PMC9469499

[5] Takeda H Hattori M Nishizawa T. Structural basis for ion selectivity revealed by high-resolution crystal structure of Mg2+ channel MgtE. Nat Commun 2014;5:5374. 25367295 10.1038/ncomms6374 PMC4241985

[6] Ferrè S Grange JS Adams-Huet Ms B. Effect of urine pH and magnesium on calcium oxalate saturation. Magnes Res 2017;30:107–119. 29637896 10.1684/mrh.2018.0429

[7] Tarleton EK. Factors influencing magnesium consumption among adults in the United States. Nutr Rev 2018;76:526–538. 29878243 10.1093/nutrit/nuy002

[8] Leung CW Ding EL Catalano PJ. Dietary intake and dietary quality of low-income adults in the Supplemental Nutrition Assistance Program. Am J Clin Nutr 2012;96:977–988. 23034960 10.3945/ajcn.112.040014 PMC3471209

[9] Tian Z Qu S Yana C. Associations of the magnesium depletion score and magnesium intake with diabetes among US adults: an analysis of the National Health and Nutrition Examination Survey 2011-2018. Epidemiol Health 2024;46:e2024020. 38271961 10.4178/epih.e2024020 PMC11099598

[10] Emelyanov A Fedoseev G Barnes PJ. Reduced intracellular magnesium concentrations in asthmatic patients. Eur Respir J 1999;13:38–40. 10836320 10.1183/09031936.99.13103899

[11] Lu J Li H Wang S. The kidney reabsorption-related magnesium depletion score is associated with increased likelihood of abdominal aortic calcification among US adults. Nephrol Dial Transplant 2023;38:1421–1429. 35881469 10.1093/ndt/gfac218

[12] Fan L Zhu X Rosanoff A. Magnesium Depletion Score (MDS) predicts risk of systemic inflammation and cardiovascular mortality among US Adults. J Nutr 2021;151:2226–2235. 34038556 10.1093/jn/nxab138 PMC8349125

[13] Ye L Zhang C Duan Q. Association of magnesium depletion score with cardiovascular disease and its association with longitudinal mortality in patients with cardiovascular disease. J Am Heart Assoc 2023;12:e030077. 37681518 10.1161/JAHA.123.030077 PMC10547298

[14] Mathew G Agha R Albrecht J. STROCSS 2021: strengthening the reporting of cohort, cross-sectional and case-control studies in surgery. Int J Surg 2021;96:106165. 34774726 10.1016/j.ijsu.2021.106165

[15] Levey AS Stevens LA Schmid CH. A new equation to estimate glomerular filtration rate. Ann Intern Med 2009;150:604–612. 19414839 10.7326/0003-4819-150-9-200905050-00006 PMC2763564

[16] McGuire S. U.S. Department of Agriculture and U.S. Department of Health and Human Services, Dietary Guidelines for Americans, 2010. Adv Nutr, 7th Edition. Washington, DC: U.S. Government Printing Office, 2011; 2011;2:293–294. 22332062 10.3945/an.111.000430 PMC3090168

[17] Liebman M Costa G. Effects of calcium and magnesium on urinary oxalate excretion after oxalate loads. J Urol 2000;163:1565–1569. 10751889

[18] Negri AL Spivacow FR. Kidney stone matrix proteins: role in stone formation. World J Nephrol 2023;12:21–28. 37035509 10.5527/wjn.v12.i2.21 PMC10075018

[19] Li Q Krieger NS Yang L. Magnesium decreases urine supersaturation but not calcium oxalate stone formation in genetic hypercalciuric stone-forming rats. Nephron 2024;1:1–7. 10.1159/000534495 PMC11219255 38262368

[20] Kharitonova M Iezhitsa I Zheltova A. Comparative angioprotective effects of magnesium compounds. J Trace Elem Med Biol 2015;29:227–234. 25127069 10.1016/j.jtemb.2014.06.026

[21] Almousa LA Salter AM Langley-Evans SC. Magnesium deficiency heightens lipopolysaccharide-induced inflammation and enhances monocyte adhesion in human umbilical vein endothelial cells. Magnes Res 2018;31:39–48. 30398154 10.1684/mrh.2018.0436

[22] Grases F Rodriguez A Costa-Bauza A. Efficacy of mixtures of magnesium, citrate and phytate as calcium oxalate crystallization inhibitors in urine. J Urol 2015;194:812–819. 25818031 10.1016/j.juro.2015.03.099

[23] Kato Y Yamaguchi S Yachiku S. Changes in urinary parameters after oral administration of potassium-sodium citrate and magnesium oxide to prevent urolithiasis. Urology 2004;63:7–11; discussion11-2. 14751336 10.1016/j.urology.2003.09.057

[24] Rylander R Remer T Berkemeyer S. Acid-base status affects renal magnesium losses in healthy, elderly persons. J Nutr 2006;136:2374–2377. 16920857 10.1093/jn/136.9.2374

[25] Mahabir S Wei Q Barrera SL. Dietary magnesium and DNA repair capacity as risk factors for lung cancer. Carcinogenesis 2008;29:949–956. 18448487 10.1093/carcin/bgn043 PMC2902380

[26] Bae YJ Choi MK. Magnesium intake and its relevance with antioxidant capacity in Korean adults. Biol Trace Elem Res 2011;143:213–225. 20978866 10.1007/s12011-010-8883-y

[27] Locatelli L Fedele G Castiglioni S. Magnesium deficiency induces lipid accumulation in vascular endothelial cells via oxidative stress-the potential contribution of EDF-1 and PPARγ. Int J Mol Sci 2021;22:105. 10.3390/ijms22031050 PMC7865876 33494333

[28] Wolf FI Trapani V Simonacci M. Magnesium deficiency and endothelial dysfunction: is oxidative stress involved? Magnes Res 2008;21:58–64. 18557135

[29] La SA Lee JY Kim DH. Low magnesium levels in adults with metabolic syndrome: a meta-analysis. Biol Trace Elem Res 2016;170:33–42. 26208810 10.1007/s12011-015-0446-9

[30] Ng HY Kuo WH Tain YL. Effect of dapagliflozin and magnesium supplementation on renal magnesium handling and magnesium homeostasis in metabolic syndrome. Nutrients 2021;13:4088. 34836340 10.3390/nu13114088 PMC8625451

[31] Askari M Mozaffari H Jafari A. The effects of magnesium supplementation on obesity measures in adults: a systematic review and dose-response meta-analysis of randomized controlled trials. Crit Rev Food Sci Nutr 2021;61:2921–2937. 32654500 10.1080/10408398.2020.1790498

[32] Han M Zhang Y Fang J. Associations between dietary magnesium intake and hypertension, diabetes, and hyperlipidemia. Hypertens Res 2024;47:331–341. 37821564 10.1038/s41440-023-01439-z

[33] Toprak O Sari Y Koç A. The impact of hypomagnesemia on erectile dysfunction in elderly, non-diabetic, stage 3 and 4 chronic kidney disease patients: a prospective cross-sectional study. Clin Interv Aging 2017;12:437–444. 28280316 10.2147/CIA.S129377 PMC5340248

[34] Al Wadee Z Ooi SL Pak SC. Serum magnesium levels in patients with obstructive sleep apnoea: a systematic review and meta-analysis. Biomedicines 2022;10:2273. 36140382 10.3390/biomedicines10092273 PMC9496273

[35] Lee CY Lee CL. Comparison of the improvement effect of deep ocean water with different mineral composition on the high fat diet-induced blood lipid and nonalcoholic fatty liver disease in a mouse model. Nutrients 2021;13:1732. 34065270 10.3390/nu13051732 PMC8160870

[36] Hoffmann J Storer RJ Park JW. N-Methyl-d-aspartate receptor open-channel blockers memantine and magnesium modulate nociceptive trigeminovascular neurotransmission in rats. Eur J Neurosci 2019;50:2847–2859. 31009120 10.1111/ejn.14423 PMC7611086

[37] Ju SY Choi WS Ock SM. Dietary magnesium intake and metabolic syndrome in the adult population: dose-response meta-analysis and meta-regression. Nutrients 2014;6:6005–6019. 25533010 10.3390/nu6126005 PMC4277012

[38] Schwalfenberg GK Genuis SJ. The importance of magnesium in clinical healthcare. Scientifica (Cairo) 2017;2017:4179326. 29093983 10.1155/2017/4179326 PMC5637834

[39] Nakashima A Ohkido I Yokoyama K. Proton pump inhibitor use and magnesium concentrations in hemodialysis patients: a cross-sectional study. PLoS One 2015;10:e0143656. 26618538 10.1371/journal.pone.0143656 PMC4664382

[40] Simmons KE Nair HR Phadke M. Risk factors for common kidney stones are correlated with kidney function independent of stone composition. Am J Nephrol 2023;54:329–336. 37253348 10.1159/000531046

[41] Apostol A Apostol R Ali E. Cerebral spinal fluid and serum ionized magnesium and calcium levels in preeclamptic women during administration of magnesium sulfate. Fertil Steril 2010;94:276–282. 19324346 10.1016/j.fertnstert.2009.02.024

[42] Si Hassen W Castetbon K Cardon P. Socioeconomic indicators are independently associated with nutrient intake in French adults: a DEDIPAC study. Nutrients 2016;8:158. 26978393 10.3390/nu8030158 PMC4808886

[43] Daponte-Codina A Knox EC Mateo-Rodriguez I. Gender and social inequalities in awareness of coronary artery disease in European countries. Int J Environ Res Public Health 2022;19:1388. 35162415 10.3390/ijerph19031388 PMC8835179

[44] Smith DM Cummins S Taylor M. Neighbourhood food environment and area deprivation: spatial accessibility to grocery stores selling fresh fruit and vegetables in urban and rural settings. Int J Epidemiol 2010;39:277–284. 19491142 10.1093/ije/dyp221

[45] Vart P Gansevoort RT Crews DC. Mediators of the association between low socioeconomic status and chronic kidney disease in the United States. Am J Epidemiol 2015;181:385–396. 25731886 10.1093/aje/kwu316 PMC4425833

[46] Richardson AS Arsenault JE Cates SC. Perceived stress, unhealthy eating behaviors, and severe obesity in low-income women. Nutr J 2015;14:122. 26630944 10.1186/s12937-015-0110-4 PMC4668704

[47] Coe FL Worcester EM Evan AP. Idiopathic hypercalciuria and formation of calcium renal stones. Nat Rev Nephrol 2016;12:519–533. 27452364 10.1038/nrneph.2016.101 PMC5837277

[48] Taylor EN Curhan GC. Dietary calcium from dairy and nondairy sources, and risk of symptomatic kidney stones. J Urol 2013;190:1255–1259. 23535174 10.1016/j.juro.2013.03.074 PMC4393945

[49] Deng X Song Y Manson JE. Magnesium, vitamin D status and mortality: results from US National Health and Nutrition Examination Survey (NHANES) 2001 to 2006 and NHANES III. BMC Med 2013;11:187. 23981518 10.1186/1741-7015-11-187 PMC3765911

[50] Hess B Jost C Zipperle L. High-calcium intake abolishes hyperoxaluria and reduces urinary crystallization during a 20-fold normal oxalate load in humans. Nephrol Dial Transplant 1998;13:2241–2247. 9761503 10.1093/ndt/13.9.2241

[51] Tallman JE Stone BV Sui W. Association between obstructive sleep apnea and 24-h urine chemistry risk factors for urinary stone disease. Urolithiasis 2023;51:46. 36881138 10.1007/s00240-023-01421-x

[52] Cho ST Jung SI Myung SC. Correlation of metabolic syndrome with urinary stone composition. Int J Urol 2013;20:208–213. 23020870 10.1111/j.1442-2042.2012.03131.x

