# Supplementary material for: Association between magnesium depletion score and the prevalence of kidney stones in the low primary income ratio: a cross-sectional study of NHANES 2007–2018
Source: Int J Surg. 2024 Jun 14;110(12):7636–46. doi: 10.1097/JS9.0000000000001822 (PMC11634088; doi:10.1097/JS9.0000000000001822)
Supplement: SUPPLEMENTARY MATERIAL [file js9-110-7636-s001.docx]

**Table S2 Association between MgDS and the prevalence of kidney stones for the low PIR with the two-day water intake adjusted.**

| **Variables (%)** | **Non-adjusted model*** | | **Minimally-adjusted model**** | | **Fully-adjusted model***** | |
| --- | --- | --- | --- | --- | --- | --- |
|  | **OR (95%CI)** | **P** | **OR (95%CI)** | **P** | **OR (95%CI)** | **P** |
| **MgDS** | 1.341 (1.249, 1.439) | <0.001 | 1.202 (1.102, 1.311) | <0.001 | 1.121 (1.017, 1.236) | 0.022 |
| **Categories of MgDS** |  |  |  |  |  |  |
| 0 | Ref |  | Ref |  | Ref |  |
| 1 | 1.366 (1.136, 1.643) | <0.001 | 1.092 (0.895, 1.332) | 0.385 | 1.147 (0.926, 1.421) | 0.210 |
| 2 | 1.887 (1.517, 2.347) | <0.001 | 1.374 (1.069, 1.767) | 0.013 | 1.232 (0.935, 1.624) | 0.138 |
| ≥3 | 2.479 (1.916, 3.207) | <0.001 | 1.809 (1.344, 2.433) | <0.001 | 1.409 (1.007, 1.971) | 0.045 |

CI: confidence interval, OR: odds ratio

*Non-adjusted model adjusts for none. 
** Minimally adjusted model adjusts for age, race. 
*** Fully adjusted model adjusts for age, gender, body mass index, race, education, marital, alcohol, smoke, diabetes, hypertension, cardiovascular disease, stroke, energy intake, healthy eating index-2015, sedentary time, vigorous activity, moderate activity, water intake, calcium intake, magnesium intake, fiber intake, fat intake.
