# Supplementary material for: Association between magnesium depletion score and the prevalence of kidney stones in the low primary income ratio: a cross-sectional study of NHANES 2007–2018
Source: Int J Surg. 2024 Jun 14;110(12):7636–46. doi: 10.1097/JS9.0000000000001822 (PMC11634088; doi:10.1097/JS9.0000000000001822)
Supplement: SUPPLEMENTARY MATERIAL [file js9-110-7636-s002.docx]

**Table S3 Association between MgDS and the recurrence of kidney stones for the low PIR with the two-day water intake adjusted.**

| **Variables (%)** | **Non-adjusted model*** | | **Minimally-adjusted model**** | | **Fully-adjusted model***** | |
| --- | --- | --- | --- | --- | --- | --- |
|  | **OR (95%CI)** | **P** | **OR (95%CI)** | **P** | **OR (95%CI)** | **P** |
| **MgDS** | 0.947 (0.773, 1.161) | 0.602 | 1.017 (0.787, 1.314) | 0.899 | 1.110 (0.819, 1.504) | 0.500 |
| **Categories of MgDS** |  |  |  |  |  |  |
| 0 | Ref |  | Ref |  | Ref |  |
| 1 | 1.350 (0.754, 2.418) | 0.313 | 1.549 (0.813, 2.949) | 0.183 | 1.726 (0.823, 3.622) | 0.149 |
| 2 | 1.165 (0.616, 2.202) | 0.639 | 1.572 (0.729, 3.387) | 0.249 | 2.270 (0.955, 5.398) | 0.064 |
| ≥3 | 0.850 (0.416, 1.737) | 0.656 | 1.160 (0.486, 2.766) | 0.739 | 1.390 (0.485, 3.979) | 0.540 |

CI: confidence interval, OR: odds ratio

*Non-adjusted model adjusts for none. 
** Minimally adjusted model adjusts for age, race. 
*** Fully adjusted model adjusts for age, gender, body mass index, race, education, marital, alcohol, smoke, diabetes, hypertension, cardiovascular disease, stroke, energy intake, healthy eating index-2015, sedentary time, vigorous activity, moderate activity, water intake, calcium intake, magnesium intake, fiber intake, fat intake.
