# Supplementary material for: Association between magnesium depletion score and the prevalence of kidney stones in the low primary income ratio: a cross-sectional study of NHANES 2007–2018
Source: Int J Surg. 2024 Jun 14;110(12):7636–46. doi: 10.1097/JS9.0000000000001822 (PMC11634088; doi:10.1097/JS9.0000000000001822)
Supplement: SUPPLEMENTARY MATERIAL [file js9-110-7636-s004.docx]

**Table S1 Characteristics of participants with low PIR by categories of the kidney stones: NHANES 2007-2018.**

| **Variables** | **All**  **(n=7,600)** | **Kidney stones** | | **P-value** |
| --- | --- | --- | --- | --- |
|  |  | **No**  **(n=6,874)** | **Yes**  **(n=726)** |  |
| **MgDS (mean ± SD)** | 0.79 ± 0.97 | 0.76 ± 0.95 | 1.07 ± 1.10 | <0.001 |
| 0 (%) | 50.18 | 51.37 | 38.98 |  |
| 1 (%) | 29.33 | 29.23 | 30.30 |  |
| 2 (%) | 13.42 | 12.89 | 18.46 |  |
| ≥3 (%) | 7.07 | 6.52 | 12.26 |  |
| **Age (years, mean ± SD)** | 47.84 ± 17.87 | 47.24 ± 17.92 | 53.44 ± 16.38 | <0.001 |
| 20-34 (%) | 28.87 | 30.36 | 14.74 |  |
| 35-49 (%) | 25.18 | 24.88 | 28.10 |  |
| 50-64 (%) | 24.49 | 24.02 | 28.93 |  |
| ≥65 (%) | 21.46 | 20.74 | 28.24 |  |
| **PIR (mean ± SD)** | 0.79 ± 0.35 | 0.79 ± 0.35 | 0.82 ± 0.34 | 0.026 |
| **BMI (kg/m2, mean ± SD)** | 29.87 ± 7.63 | 29.77 ± 7.66 | 30.76 ± 7.29 | 0.001 |
| <25 (%) | 26.94 | 27.69 | 19.83 |  |
| ≥25 and <30 (%) | 31.11 | 31.04 | 31.84 |  |
| ≥30 (%) | 41.94 | 41.27 | 48.32 |  |
| **Gender (%)** |  |  |  | 0.167 |
| Female | 55.32 | 55.57 | 52.89 |  |
| Male | 44.68 | 44.43 | 47.11 |  |
| **Race (%)** |  |  |  | <0.001 |
| Mexican American | 19.92 | 20.28 | 16.53 |  |
| Other Hispanic | 37.11 | 35.61 | 51.24 |  |
| Non-Hispanic white | 23.00 | 24.08 | 12.81 |  |
| Non-Hispanic black | 12.03 | 12.00 | 12.26 |  |
| Other races | 7.95 | 8.03 | 7.16 |  |
| **Education (%)** |  |  |  | 0.137 |
| Less than 9th grade | 16.63 | 16.42 | 18.60 |  |
| 9-11th grade | 22.28 | 22.27 | 22.31 |  |
| High school graduate | 26.58 | 26.83 | 24.24 |  |
| Some college | 26.35 | 26.15 | 28.24 |  |
| College graduate or above | 8.16 | 8.33 | 6.61 |  |
| **Marital (%)** |  |  |  | <0.001 |
| Married/Living with partner | 47.01 | 46.90 | 48.07 |  |
| Divorced/Separated/Widowed | 29.86 | 29.05 | 37.47 |  |
| Never married | 23.13 | 24.05 | 14.46 |  |
| **Alcohol (%)** |  |  |  | 0.001 |
| Never | 17.74 | 17.76 | 17.59 |  |
| Former | 20.87 | 20.30 | 26.17 |  |
| Yes | 61.39 | 61.94 | 56.24 |  |
| **Smoke (%)** |  |  |  | <0.001 |
| Never | 48.43 | 49.09 | 42.15 |  |
| Former | 21.39 | 20.88 | 26.17 |  |
| Yes | 30.18 | 30.02 | 31.68 |  |
| **Stroke (%)** |  |  |  | 0.063 |
| No | 94.58 | 94.74 | 93.09 |  |
| Yes | 5.42 | 5.26 | 6.91 |  |
| **HBP (%)** |  |  |  |  |
| No | 55.32 | 56.62 | 42.98 |  |
| Yes | 44.68 | 43.38 | 57.02 |  |
| **CVD (%)** |  |  |  | <0.001 |
| No | 86.33 | 87.49 | 75.31 |  |
| Yes | 13.67 | 12.51 | 24.69 |  |
| **Diabetes (%)** |  |  |  | <0.001 |
| No | 70.16 | 70.90 | 63.09 |  |
| Borderline | 8.46 | 8.58 | 7.30 |  |
| Yes | 21.38 | 20.51 | 29.61 |  |
| **Water intake (%)** | 1019.50 ± 1215.76 | 1014.94 ± 1199.15 | 1062.65 ± 1363.19 | 0.315 |
| Inadequate | 85.88 | 86.03 | 84.44 |  |
| Adequate | 14.12 | 13.97 | 15.56 |  |
| **Moderate activity** |  |  |  | 0.098 |
| No | 68.93 | 68.64 | 71.63 |  |
| Yes | 31.07 | 31.36 | 28.37 |  |
| **Vigorous activity** |  |  |  | <0.001 |
| No | 85.18 | 84.64 | 90.36 |  |
| Yes | 14.82 | 15.36 | 9.64 |  |
| **Sedentary time (%)** | 323.69 ± 199.22 | 323.18 ± 199.16 | 328.54 ± 199.84 | 0.492 |
| <150 mins/day | 20.50 | 20.57 | 19.78 |  |
| ≥150 mins/day | 79.50 | 79.43 | 80.22 |  |
| **Calcium intake (%)** | 885.31 ± 517.24 | 889.22 ± 520.70 | 848.27 ± 482.02 | 0.042 |
| <300 mg/day | 5.84 | 5.80 | 6.20 |  |
| ≥300 mg/day | 94.16 | 94.20 | 93.80 |  |
| **Fiber intake (mg/day, mean ± SD)** | 15.51 ± 9.18 | 15.63 ± 9.28 | 14.43 ± 8.11 | <0.001 |
| **Fat intake (mg/day, mean ± SD)** | 73.54 ± 39.84 | 73.76 ± 39.88 | 71.49 ± 39.40 | 0.144 |
| **Magnesium intake (mg/day, mean ± SD)** | 269.86 ± 128.14 | 271.50 ± 129.32 | 254.30 ± 115.29 | <0.001 |
| **Energy intake (kcal, mean ± SD)** | 2063.28 ± 1080.28 | 2072.78 ± 1080.11 | 1973.31 ± 1078.53 | 0.018 |
| **HEI-2015 (mean ±SD)** | 48.69 ± 13.44 | 48.81 ± 13.46 | 47.63 ± 13.16 | 0.025 |

* Mean + SD for continuous variables, and P value was calculated by weighted t test. % for categorical variables, and P value was calculated by weighted chi-square test. SD=Standard Deviation, MgDS = Magnesium Depletion Score, BMI = Body Mass Index, PIR = Poverty Income Ratio, CVD = Cardiovascular Disease, HBP = Hypertension, HEI-2015 Index= Healthy Eating Index-2015.
